# Tissue-based skin prick test extracts from Atlantic salmon containing occupationally relevant allergens

**DOI:** 10.3389/falgy.2025.1525012

**Published:** 2025-06-23

**Authors:** Inga Elda, Miriam Grgic, Klara Stensvåg, Berit Bang

**Affiliations:** ^1^ Department of Occupational and Environmental Medicine, University Hospital North Norway, Tromsø, Norway; ^2^ Department of Medical Biology, University of Tromsø—The Arctic University of Norway, Tromsø, Norway; ^3^ The Norwegian College of Fishery Science, Faculty of Biosciences, Fisheries and Economics, University of Tromsø—The Arctic University of Norway, Tromsø, Norway

**Keywords:** allergen extracts, occupational allergy, salmon allergy, salmon processing, skin prick testing, thermolabile allergen

## Abstract

**Introduction:**

Diagnosis of salmon allergy often includes skin prick testing using commercially available extracts. Multiple studies have shown that these may contain highly variable amounts of important allergens. In this study we aimed to produce skin prick test extracts containing salmon allergens relevant to the occupational exposure situation. The extracts were characterized with respect to the presence of known salmon allergens. The effect of heat-treatment on the antibody-binding of allergens was also assessed.

**Method:**

Allergenic proteins were extracted from muscle, skin, and outer mucus coating from Atlantic salmon (*Salmo salar*). Electrophoresis was used for protein separation with subsequent silver staining and immunoblotting for the detection of known allergenic proteins with antibody-binding activity to commercially available antibodies. The identity of allergens was confirmed by mass spectrometry.

**Results:**

Our extraction methods were successful in detecting the allergenic proteins aldolase, collagen, enolase, and parvalbumin from muscle, skin, and outer mucus coating from Atlantic salmon. Our work also demonstrates that these allergens have various tolerance towards heating.

**Conclusion:**

By using multiple fish tissues for extraction, we were able to produce skin prick test extracts containing allergens of presumed relevance in diagnosis of work-related sensitization.

## 1 Introduction

Aquaculture and seafood industries are increasing on a global basis, as seafood is becoming more important as a protein source in the human diet (1). At the same time, it has been found that seafood processing can cause occupational allergy and asthma (2), with a prevalence of occupational asthma between 2% and 8% among workers in the fish industry (3). Studies have shown that Norwegian salmon industry workers are exposed to bioaerosols (4–6) and are at risk of respiratory impairment (4–8). An ongoing research project (SHInE) (9), aims to identify interventions that can reduce exposure to bioaerosols in Norwegian salmon processing plants. Bioaerosols in salmon processing environments have been found to contain biologically active proteins, such as allergens and enzymes (4–6), which can cause symptoms in the upper or lower respiratory tract, as well as skin symptoms (10). There are currently seven registered allergens from Atlantic salmon (*Salmo salar*): beta-parvalbumin 1, beta-enolase, aldolase A, tropomyosin, collagen alpha, creatine kinase, and triosephosphate isomerase (11). In this article, the focus is on the four most prevalent allergens: parvalbumin, enolase, aldolase, and collagen.

Parvalbumin is known as the major fish allergen, first identified as an allergen in cod (12), and later in other species such as tuna (13), carp (14), mackerel (15) and salmon (16). It is an acidic, highly stable, heat-resistant and Ca^2+^-binding muscle protein with a molecular weight of approximately 10–12 kDa (17). Parvalbumin belongs to the EF-hand superfamily of proteins that contains a characteristic cation binding helix-loop-helix structural motif (18). Oligomeric forms of higher molecular weights have been found in Atlantic cod (19). Collagen is a triple helix made up of alpha chains (20) first identified as an allergen in a study using sera from patients who had a previous history of fish allergy (21). Further studies have found the allergen to be thermostable (22). Gelatine is the hydrolyzed form of collagen, which is a multifunctional molecule used in both pharmaceutical, drug, and food industries. As collagen, gelatine might also act as an allergen as reported by Sakaguchi et al. (23). A severe case of anaphylaxis has also been described in a patient which ingested marshmallows containing fish gelatine (24). Enolase (50 kDa) is a metalloenzyme catalyzing the ninth step in the glycolytic pathway, depending on a magnesium ion (Mg^2+^) for its function. Aldolase (40 kDa) is also an enzyme in the glycolysis, catalyzing the fourth step. Both enzymes are present in high amounts in fish muscle, thermolabile, and were identified as a fish allergen in a study where fish allergic patients displayed IgE binding to aldolase and enolase, but not to the major fish allergen parvalbumin (25).

Generally, the diagnosis of fish allergy involves a combination of clinical evaluation, skin prick testing (SPT), and specific IgE testing (sIgE). Other approaches include oral food challenges (26), with the double-blinded placebo-controlled food challenge (DBPCFC) being the gold standard for food allergy and specific inhalation challenge (SIC) being the gold standard for occupational allergy (27). However, due to the time-consuming and costly nature of both these methods simpler methods are often considered sufficient. SPT is a rapid and relatively non-invasive tool that can be used to detect sensitization to allergens. The test is performed by placing a droplet of the test extract on the patient's forearm and pricking it into the skin with a sterile lancet. After 15 min, the wheal size is measured, with a size ≥3 mm considered as a positive test result. The clinical relevance of a positive test result should be interpreted along with previous medical history. A positive test result does not necessarily reflect clinical allergy, as false positives may occur. Standardized allergen extracts would contribute to improve positive as well as negative predictive values of SPTs. To our knowledge, the predictive values of SPT for occupational salmon allergy is not previously reported. With this method, it is most common to use commercially available test extracts, that contain allergens from the source of interest. Several previous studies have demonstrated that such extracts, including fish extracts, may have a highly variable content of clinically relevant allergens (28–30). To help promote the diagnosis of fish allergy, standardized methods for making test extracts are needed. Samples from various fish tissues are sometimes used in prick-to-prick protocols, but these are even less standardized with respect to the presence of important allergens, as variations may occur within the tissue that is being used (31). Well-characterized skin prick extracts can provide a much-needed specificity in the performance of SPT, as this tool is used daily in both small and highly specialized allergy clinics. Future research should aim to produce extracts of pharmaceutical standard to avoid batch-to-batch variations. The use of well-characterized skin prick test extracts from several types of fish tissue will provide more specific information. In combination with serum analyses such as sIgE (ImmunoCAP) and symptom profiling, it will improve personalized guidance on allergen avoidance, as well as reduce unnecessary restrictive dietary behavior or occupational choices. Such information is also a prerequisite for the future development of recombinant allergens for component-resolved diagnosis (CRD) and allergen immunotherapy (AIT) protocols (32–34). With the work presented here, we suggest methods for making mucus, muscle, and skin tissue extracts from Atlantic salmon, containing the allergens aldolase, collagen, enolase, and parvalbumin.

## 2 Materials and methods

### 2.1 Protein extraction from Atlantic salmon tissue

#### 2.1.1 Biological material

Fresh fish (Atlantic salmon, *Salmo salar*) was obtained from Tromsø Aquaculture Research Station where it was killed by percussive stunning. The fish was kept on ice during transport to the laboratory. Tissue samples were separated immediately after arrival to the lab, minimizing degradation and post-mortem growth of microorganisms. The first step was to remove the mucus by using a scraping method (35). Pieces of the skin were cut off, before the muscle was separated from the remaining parts of the fish and cut into smaller pieces. All tissue parts were aliquoted and stored at −20°C until further use.

#### 2.1.2 Muscle

Muscle tissue from salmon was thawed from −20°C. The extraction of proteins from the tissue was based on previous work, as described by Thomassen and Kamath et al. (36). An aliquot of 25–50 g of biological material was homogenized in PBS-T (1× PBS, 0.05% Tween® 20) (w/v) using a kitchen blender (BOSCH SilentMixx CNSM13). Centrifugation followed at 4,000 × g for 10 min at 4°C (Beckman Coulter Allegra® X-15R Centrifuge) and was repeated with the obtained supernatant. The remaining supernatant was subjected to centrifugation at 30,000 × g for 35 min at 4°C (Beckman Coulter Optima™ LE-80K Ultracentrifuge)^1^. The final supernatant from this stepwise centrifugation was collected and mixed with 50% glycerol to achieve 10% glycerol (v/v). It was then aliquoted, flash frozen with liquid nitrogen, and stored at −70°C until further use.

#### 2.1.3 Mucus

Extraction of mucus was based on a published study by Kumari et al. (37). Mucus was mixed with extraction buffer (0.05 M Tris-HCl, pH 7, 0.14 M NaCl) in a 1:1 (w/v) ratio and incubated at 4°C overnight. On the following day, the sample was centrifuged at 4,500 rpm for 15 min (Beckman Coulter Allegra® X-15R Centrifuge) at 4°C. The supernatant was transferred to a protein concentrator tube (Amicon Ultra-15 10 K, Merck MilliPore) and centrifuged again using the same parameters. The final supernatant was collected and mixed with glycerol (10% v/v), before it was aliquoted, and flash frozen with liquid nitrogen. Aliquots were stored at −70°C until further use.

#### 2.1.4 Skin

Fish skin was obtained from −20°C, excess muscle tissue and fat was removed, and the skin was cut into pieces of ∼1 × 1 cm. All centrifugation and incubation overnight in the following work was done at 4°C. The extraction method was based on previous literature by Wu et al. (38), Jafari et al. (39) and Barzkar et al. (40). To remove non-collagenous proteins, the skin was mixed with 0.1 M NaOH (10% w/v) and incubated for 48 h on a shaker. Next, the skin pieces were washed with deionized water until reaching a neutral pH level, tested with litmus paper. The skin pieces were then mixed with 10% butanol (1:10 v/v) for the purpose of defatting for 24 h, followed by removal of the butanol. The skin pieces were then suspended in 0.5 M acetic acid for 24 h for extraction of the acid-soluble collagen (ASC). The next day, the sample was centrifuged at 5,000 × g for 60 min (Beckman Coulter Allegra® X-15R Centrifuge). Following centrifugation, the supernatant was mixed with 5 M NaCl until a final concentration of 1.2 M NaCl, which was then left to incubate for 24–48 h. After salting out with NaCl, centrifugation at 20,000 × g for 60 min was done (Beckman Coulter Optima™ LE-80K Ultracentrifuge)^1^, before the pellet was resuspended in 0.5 M acetic acid. The precipitate was dialyzed against 0.1 M acetic acid overnight using a snakeskin dialysis membrane (10K MWCO, Thermo Scientific™). Finally, 0.1 M acetic acid was removed and replaced with 2 L of deionized water for dialysis for 68–72 h. The skin extract was then mixed with glycerol (10% v/v), aliquoted, flash frozen with liquid nitrogen, and stored at −70°C until used.

### 2.2 Characterization of proteins in tissue extracts by BCA and SDS-PAGE

The protein extracts were subjected to protein quantification, using the QuantiPro™ BCA Assay Kit (Sigma-Aldrich®) in accordance with the manufacturer's guidelines. Absorbance was read at 540 nm (Multiskan EX plate-reader, Thermo Fisher Scientific) and used to determine total protein content (µg/ml). Protein extracts from muscle, mucus, and skin were mixed 1:4 with SDS sample buffer (250 mM Tris-HCl, 10% SDS, 2% Bromophenol blue, 50% Glycerol) and separated electrophoretically, using 4%–12% Bis-Tris Gels (NuPAGE™, Invitrogen) in a Mini Gel Tank (Invitrogen). Electrophoresis was done at 120 V, 200 mA for ∼60 min. Protein bands were visualized using either the SilverQuest™ Silver Staining Kit (Thermo Fisher Scientific) or Coomassie® Brilliant blue G 250 (Sigma Aldrich).

### 2.3 Mass spectrometry (Ms/Ms)

Protein extracts from raw muscle, skin and outer mucus coating were subjected to SDS-PAGE as described above before they were stained with Coomassie brilliant blue for visualization of protein bands. Bands corresponding to the molecular weight of the monomeric form of the allergenic proteins collagen (∼110 kDa), enolase (∼50 kDa), aldolase (∼40 kDa) and parvalbumin (∼12 kDa) were cut out with a sterilized scalpel and stored at 4°C until analysis. Mass spectrometric analysis was conducted at The Proteomics and Metabolomics Core Facility (PRiME) at the Department of Medical Biology, UiT The Arctic University of Norway. Gel pieces were subjected to in-gel reduction, alkylation, and tryptic digestion using 6 ng/µl trypsin (V511A, Promega, Wisconsin, USA). OMIX C18 tips (Varian, Inc., Palo Alto, CA, USA) were used for sample cleanup and concentration. Peptide mixtures containing 0.1% formic acid were loaded onto a Thermo Fisher Scientific EASY-nLC1200 system and EASY-Spray column (C18, 2 µm, 100 Å, 50 µm, 50 cm). Peptides were fractionated using a 5%–80% acetonitrile gradient in 0.1% formic acid over 140 min at a flow rate of 0,3 μl/min. The separated peptides were analyzed using a Thermo Scientific Orbitrap Exploris 480 mass spectrometer. Data was collected in data-dependent mode using a Top20 method. The raw data were processed using the Proteome Discoverer 2.5 software. The fragmentation spectra were searched against the UniProt Salmo salar database from 2020. Peptide mass tolerances used in the search were 10 ppm, and fragment mass tolerance was 0.02 Da. Peptide ions were filtered using a false discovery rate (FDR) set to 5% for peptide identifications. Values used for the identification of proteins were coverage (%), number of peptides, number of peptide spectrum matches (PSMs), molecular weight (kDa), and accession number. Coverage was identified as the percentage of identified peptides found in the protein sequence. The number of peptides was the amount of peptide sequences identified in the protein, and PSMs were the total number of peptide spectra matching the protein sequence. Suggested molecular weight was also found in kDa, as well as an accession number for the protein.

### 2.4 Immunoblotting

The protein extracts were separated by SDS-PAGE as previously detailed, and then transferred to a nitrocellulose membrane using a Power Blotter—Semi-dry Transfer System (Thermo Fisher Scientific). Immunoblotting was then done using either the iBind Western Automated system (Thermo Fisher Scientific) following manufacturers protocol, or manually.

With the manual protocol, the membrane was first washed briefly in deionized water, and then incubated in blocking buffer (4% milk powder, 0.05% Tween® 20, 1x PBS) for 60 min at room temperature. After this, washing for 5 × 6 min with PBS-T (0.05% Tween® 20, 1x PBS) followed, before incubation with primary antibodies for 60 min was done. The primary antibodies utilized were mouse monoclonal anti-parvalbumin (Sigma-Aldrich®), rabbit polyclonal anti-collagen (Cedar Lane Labs), polyclonal rabbit anti-enolase (Aviva Systems Biology) and polyclonal rabbit anti-aldolase (Aviva Systems Biology), all diluted to a ratio of 1:2,000 with blocking buffer. Following another 5 × 6 washing with PBS-T, the membrane was incubated with secondary antibody, diluted to a ratio of 1:20,000 in blocking buffer, for 60 min. The secondary antibody for parvalbumin was goat anti-mouse IgG HRP conjugate (Bio-Rad), and for aldolase, collagen, and enolase it was goat anti-rabbit IgG HRP conjugate (Bio-Rad). Finally, the membrane was washed 5 × 6 with PBS-T again, followed by a 2 × 6 min was with 1x PBS only. Antibody-binding activity was detected using a chemiluminescent method with the SuperSignal™ West Femto Maximum Sensitivity Substrate kit (Thermo Fisher Scientific). Development of the membrane was done using ImageQuant LAS 4,000 (GE Healthcare) with the sensitivity set to high resolution and an exposure time of 2–20 min.

Briefly, with the iBind Automated system, antibodies were diluted 1:2,000 (primary) and 1:4,000 (secondary) with 1x iBind Flex Buffer. The membrane was submerged in the iBind buffer, while adding 5 ml (mini blot) or 10 ml (midi blot) buffer evenly to the iBind Flex Card. The membrane was then placed on the flex card, with protein side facing down, using a blotting roller to remove any air bubbles. The diluted antibodies and buffer were then added to the wells. Incubation of the membrane was done for 3–4 h, before development as described above followed.

### 2.5 Temperature effect on antibody-binding

The antigen-binding activity of allergenic proteins in muscle, mucus, and skin extracts was assessed at variable temperatures. Five different temperatures were used in the testing: 0°C (extract kept on ice as control), 20°C (room temperature), 60°C, 80°C, and 100°C. All samples were tested for 5 and 30 min. The temperature-treated samples were diluted 1:4 with SDS sample buffer and subjected to electrophoresis and immunoblotting as described previously.

### 2.6 Skin prick testing

Skin prick testing (SPT) was performed on salmon processing workers from the SHInE cohort (33). The testing was performed by trained clinical personnel. One commercial extract for Atlantic cod (ALK) was used. In-house extracts included raw muscle, heated muscle (100°C, 30 min), outer mucus coating and skin from Atlantic Salmon. For positive control, histamine (10 mg/ml) (ALK) was used. Due to different extraction methods for the in-house extracts, two negative controls were used: in-house negative control 1 (1x PBS, 0.05% Tween® 20, 10% glycerol) and in-house negative control 2 (0.2 M acetic acid, 10% glycerol). A droplet of extract (∼15 μl) was placed on the volar forearm and pricked into the skin using a sterile lancet. After 15 min, reactions were read by measuring the wheal size (mm). All workers signed informed consent before taking part in the study. Ethical approval was obtained from the Regional Committee for Medical Research Ethics North Norway (REK Nord No. 175081). Among 784 tested workers, we chose to include the results of four workers in the current paper. They were selected based on having individually different reactions to our in-house tissue extracts, thereby representing different sensitization profiles. We only included four workers in the current paper, to demonstrate the use of our in-house SPT extracts. In the next paper, we will include the findings of a larger study population from the SHInE study.

## 3 Results

### 3.1 Protein content in tissue extracts

**Figure 1:**
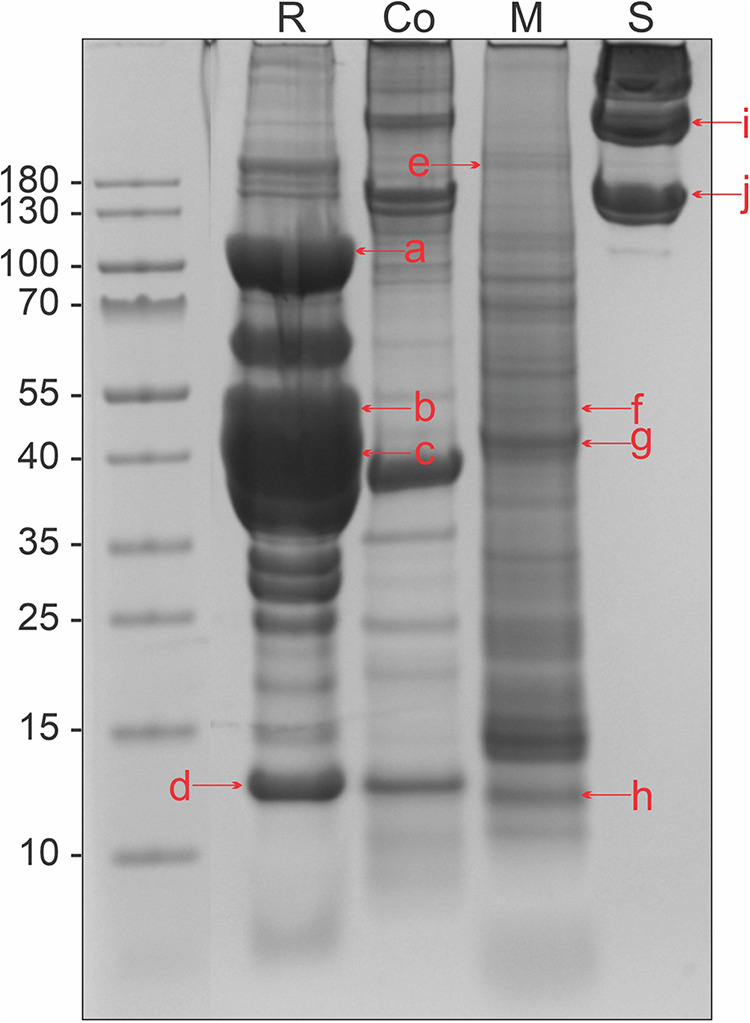
Protein content in tissue extracts from Atlantic salmon, separated by SDS-PAGE in a 4%–12% Bis-Tris gel and stained with Coomassie. R, raw muscle; Co, heated muscle (100°C 30 min); M, mucus; and S, skin. Letters in lowercase corresponds to bands that were analyzed using mass spectrometry, matching the monomeric form of the four allergens collagen (∼110 kDa): a, e, i, and j, enolase (∼50 kDa): b and f, aldolase (∼40 kDa): c and g, and parvalbumin (∼12 kDa): d and h. Extracts used were RM1, M1, CM1, and S1 from Supplementary Table S1.

With the BCA method, we analyzed the protein content of four different tissue extracts from Atlantic salmon: raw and cooked muscle, mucus, and skin (Supplementary Table S1). The raw muscle extract had a protein content with a range of 21 μg/ml to 12 920 μg/ml. In the mucus and skin extracts, the protein content was found to be 197–6,524 μg/ml and 58–6,577 μg/ml, respectively. The broad range of protein content was due to different centrifugation methods available at the time of extractions, as referred to in the methods section. The qualitative composition of the different tissue extracts was found by SDS-PAGE and subsequent Coomassie staining, revealing varying protein content in the extracts (Figure 1). Based on visual inspection, the highest abundance of protein bands was found in the raw extract. The cooked muscle extract revealed that degradation of proteins had occurred, as this extract had fewer protein bands compared to the raw muscle extract. The mucus extract displayed relatively few intense protein bands, as compared to the raw and cooked muscle extract. In the skin extract, intense protein bands were present only at relatively high molecular weights (>∼130 kDa).

### 3.2 Mass spectrometry

The raw muscle, mucus, and skin tissue extracts were analyzed with mass spectrometry for the identification of the four allergens collagen, enolase, aldolase, and parvalbumin (Supplementary Tables S2–S4). Bands were cut out from a protein gel, at molecular weights corresponding to the approximate monomeric form of the allergen (Figure 1), referred to as lowercase letters in the figure. Aldolase and enolase were found in both mucus and muscle tissue extracts, in several SDS-PAGE protein bands. Collagen was consistently identified in all three extracts, whereas parvalbumin was only present in the raw muscle extract (Table 1).

**Table 1:** Salmon allergens identified in mucus, raw muscle, and skin tissue extracts, from SDS-PAGE bands corresponding to the monomeric form of the allergens of interest.

Tissue	Mw of SDS-PAGE band (kDa)	Identifier in protein gel and western blot	Identified protein(s)
Raw muscle	∼110	a	Aldolase, enolase, collagen
	∼50	b	Enolase, aldolase, parvalbumin
	∼40	c	Aldolase, enolase, collagen
	∼12	d	Parvalbumin, aldolase, enolase
Mucus	∼180	e	Collagen
	∼50	f	Enolase, aldolase, collagen
	∼40	g	Aldolase, enolase, collagen
	∼12	h	Collagen, enolase
Skin	>180	i	Collagen
	∼180	j	Collagen

### 3.3 Qualitative allergen content in protein extracts

#### 3.3.1 Raw muscle

**Figure 2:**
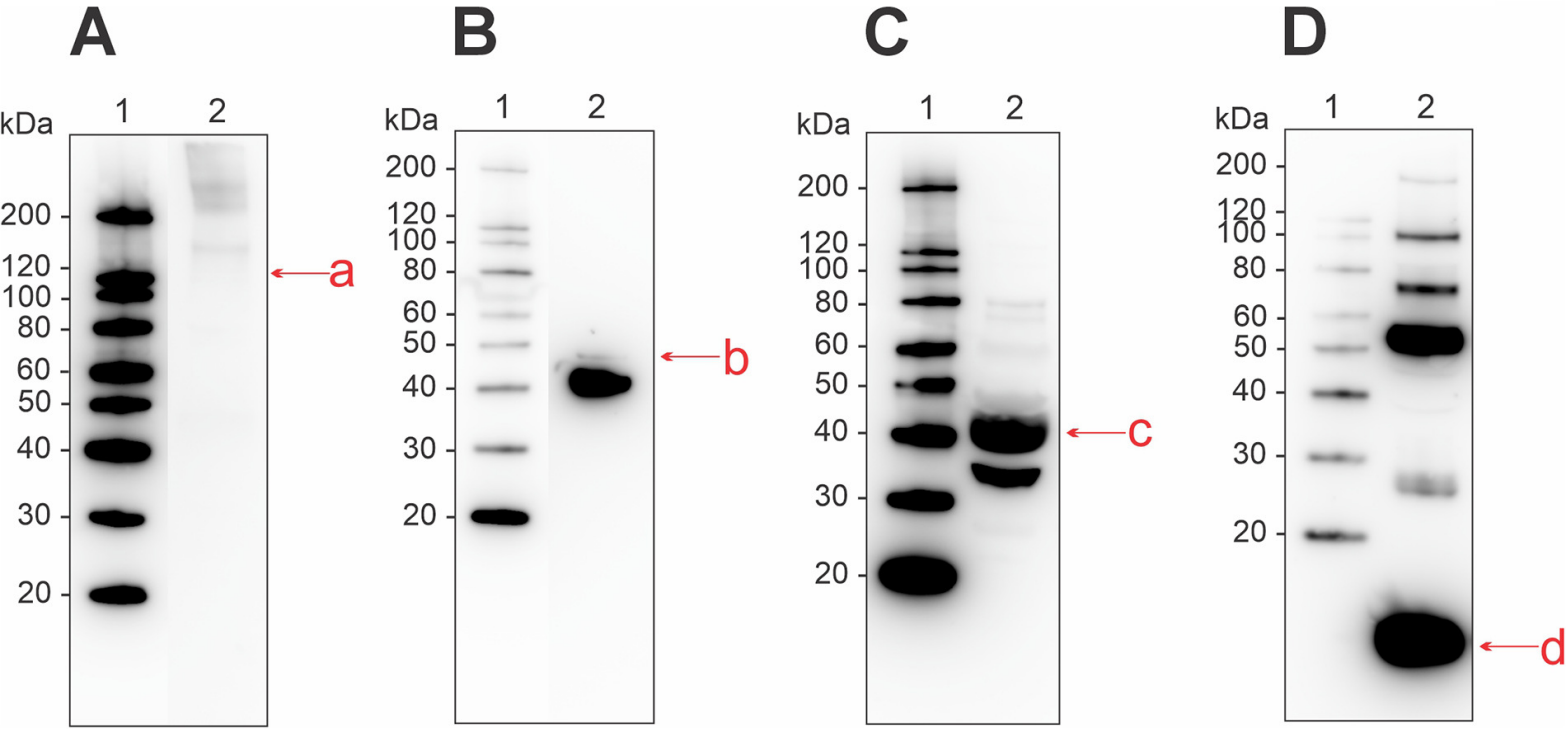
Western blot for detection of allergenic proteins in a raw muscle tissue extract from Atlantic salmon, using commercial antibodies for **(A)** collagen, **(B)** enolase, **(C)** aldolase, and **(D)** parvalbumin. Letters in lowercase corresponds to bands that were analyzed using mass spectrometry, matching the monomeric form of the four allergens. Extract used was RM2 from Supplementary Table S1.

With immunoblotting and commercial antibodies, we identified the four allergens collagen, enolase, aldolase, and parvalbumin to be present in the raw muscle extract (Figure 2). Collagen displayed weak antibody-binding in protein bands at higher molecular weights (>120 kDa) (Figure 2A). For enolase, a strong band corresponding to the monomeric form was present at ∼40 kDa, as well as a weaker band at ∼47 kDa (Figure 2B). Aldolase was found as a monomer at ∼40 kDa and as a potential cleavage product at ∼35 kDa (Figure 2C). Parvalbumin displayed strong antibody-binding activity at the monomeric form (∼12 kDa), and as suspected oligomers (25–180 kDa) (Figure 2D).

#### 3.3.2 Mucus

**Figure 3:**
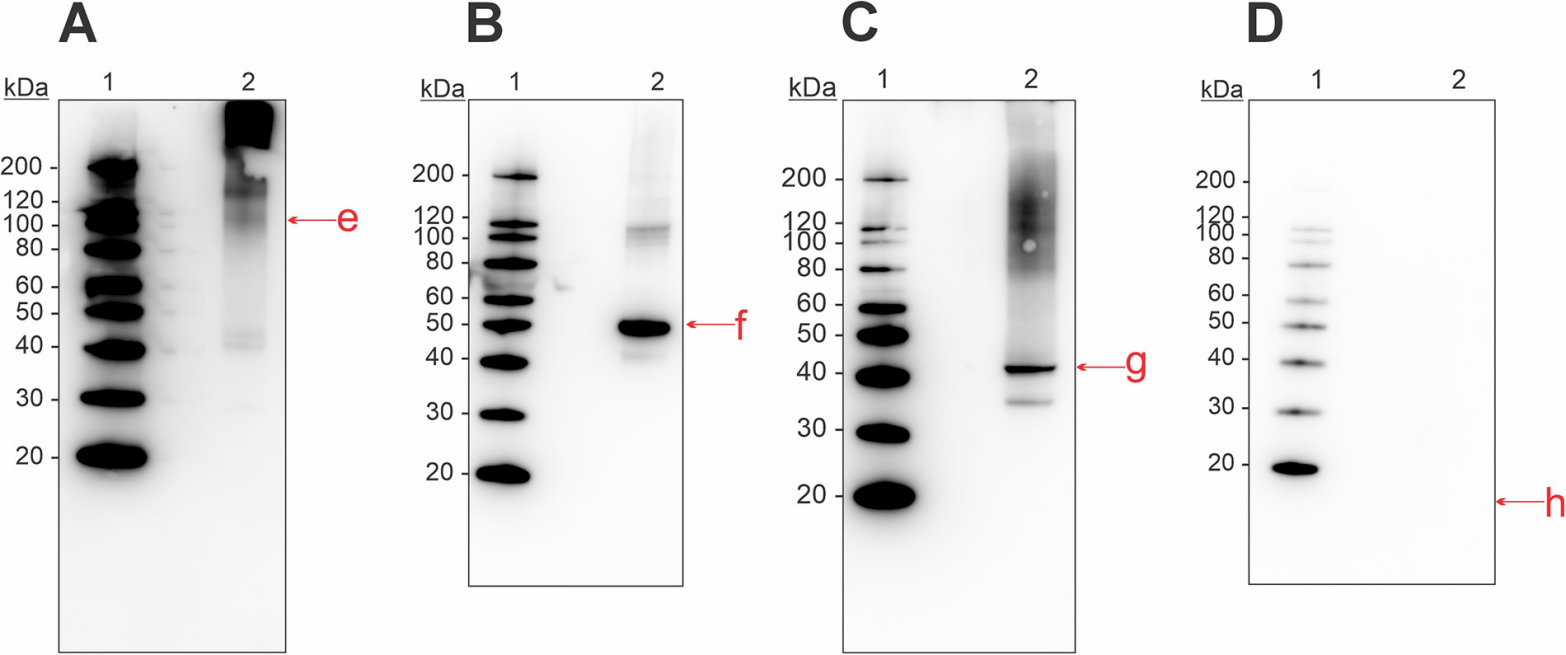
Western blot for detection of allergenic proteins in a mucus tissue extract from Atlantic Salmon, using commercial antibodies for **(A)** collagen, **(B)** enolase, **(C)** aldolase, and **(D)** parvalbumin. Letters in lowercase corresponds to bands that were analyzed using mass spectrometry, matching the monomeric form of the four allergens. Extract used was M2 from Supplementary Table S1.

In the mucus extract we identified the presence of aldolase, enolase, and collagen through immunoblotting and commercial antibodies (Figure 3). Antibody-binding activity for collagen was found at molecular weights higher than ∼100 kDa (Figure 3A). One weak band was also present around 40 kDa, which might be due to degradation. Enolase was present in both monomeric (50 kDa) and dimeric (100 kDa) forms (Figure 3B). Aldolase had binding activity in its monomeric form at 40 kDa, and at ∼35 kDa (Figure 3C). Additionally, some binding occurred around 120 kDa, suggesting oligomerization. Despite multiple attempts, we were not able to identify the presence of parvalbumin in the mucus extract (Figure 3D).

#### 3.3.3 Skin

**Figure 4:**
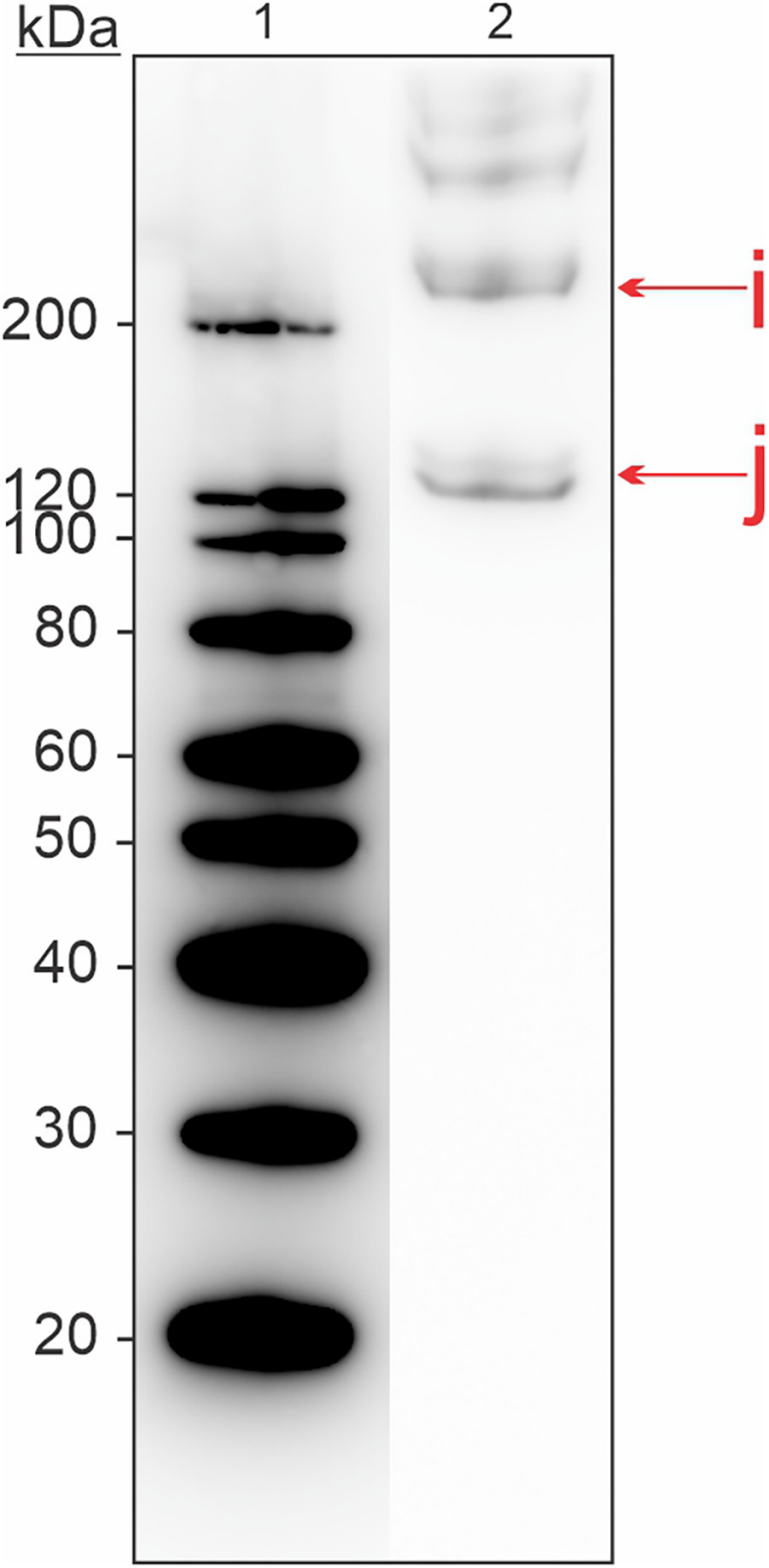
Western blot for detection of allergenic proteins in a skin tissue extract from Atlantic salmon, using a commercial antibody for collagen. Letters in lowercase corresponds to bands that were analyzed using mass spectrometry. Extract used was S2 from Supplementary Table S1.

The skin extract was analyzed with immunoblotting and a commercial antibody for collagen (Figure 4). Bands were present at molecular weights ∼120, ∼200, and >200 kDa, indicating the presence of collagen in various isoforms in this tissue extract.

### 3.4 Temperature stability of salmon allergens in the protein extracts

Protein extracts from muscle, mucus, and skin were exposed to various temperatures and analyzed with SDS-PAGE (Supplementary Figure S1). Immunoblotting with commercial antibodies for the four salmon allergens collagen, enolase, aldolase, and parvalbumin was done to assess their heat stability in the different protein extracts (Supplementary Figures S2–S4). Table 2 displays how the allergens aldolase, collagen, enolase, and parvalbumin respond to heating in muscle-, skin-, and mucus protein extracts. Aldolase and enolase in the heated muscle extract displayed low stability towards heating, as protein bands had disappeared at 60°C for both 5 and 30 min, respectively. The protein band for aldolase was weakened already at room temperature, 30 min. Parvalbumin in the heated muscle extract showed more stability towards heating, compared to aldolase and enolase. The protein band corresponding to the monomeric form of parvalbumin (∼12 kDa) started to weaken at 60°C, 30 min. After heating at 100°C for 5 min the relative band intensity is stronger but heating at 100°C for 30 min causes the band to disappear. In the heated skin extract, collagen started to degrade when heated at 80°C for 30 min, but was stable at 100°C, 5 min. More degradation occurred at prolonged heating; 100°C for 30 min. Aldolase and, interestingly, collagen are both temperature sensitive in the heated mucus extract, with their respective protein bands disappearing already at 60°C, 5 min. The protein band for enolase disappeared after heating at 60°C for 30 min.

**Table 2:** Heat stability of salmon allergens in muscle, skin, and mucus protein extracts. The protein extracts were exposed to five different temperatures at two different time points (5 and 30 min). C, change in antibody-binding; D, disappearance of antibody-binding; -, no change in antibody-binding; empty cell, no antibody-binding. Based on Supplementary Figures S2-S4.

Heated extract	Allergen	Mw of protein band (kDa)	Antibody-binding				
			Temperature (°C)				
			0	RT	60	80	100
Muscle	Aldolase	∼40	-	C (30)	D (5)		
	Enolase	∼50	-	-	C (5)/D (30)		
	Parvalbumin	∼12	-	-	C (5)	-	D (30)
Skin	Collagen	∼120	-	-	-	-	-
Mucus	Aldolase	∼40	-	C (5)	D (30)	-	-
	Collagen	>200	-	-	C (5)/D (30)		
	Enolase	∼50	-	-	C (5)/D (30)		

### 3.5 Individual skin prick test reactions

Four workers from the SHInE-study were included in this study, to exhibit different sensitization profiles in salmon processing workers. The workers were tested using both in-house tissue extracts, as well as a commercial extract from cod. Worker 1, 3, and 4 all had a reaction on the raw muscle extract (Table 3). Worker 2 had a reaction on the in-house mucus extract only. Worker 3 reacted on all four in-house extracts, but not the commercial cod extract. Worker 4 reacted on both raw and heated muscle extracts, as well as the commercial cod extract. None of the four workers presented here had a reaction on the negative controls. In Europe, there are few commercially available SPT extracts for salmon. The few extracts we were able to obtain at the time of investigation, did not contain the relevant allergens in detectable amounts.

**Table 3:** Four salmon processing workers' reaction on skin prick testing using both in-house tissue extracts and a commercial cod extract. The results are shown as either negative (−) or positive (+), with a positive test being defined as a wheal size ≥3 mm read 15 min after the test was performed. All workers had a reaction on a positive control, and no reaction on negative controls.

Worker no.	In-house				Commercial
	Raw muscle	Heated muscle^a^	Mucus	Skin	Cod^b^
1	+	-	+	-	-
2	-	-	+	-	-
3	+	+	+	+	-
4	+	+	-	-	+

^a^ Heated at 100°C for 30 min.

^b^ Commercial SPT extract for Atlantic Cod (ALK).

## Discussion

The present study was conducted to make protein extracts from different salmon tissues, containing the four known fish allergens collagen, enolase, aldolase, and parvalbumin. The extracts were further tested on Norwegian salmon processing workers, using the SPT. Previous studies have shown that commercially available SPT extracts have variable levels of important allergens, and allergens of special relevance to the occupational setting are often lacking (28). Workers in the salmon industry are exposed to the outer mucus coating of salmon, as well as the skin and muscle tissue. Thus, the present study aimed to customize extraction protocols for tissues relevant in the occupational exposure context. The extracts were characterized with respect to the presence of the four salmon allergens: aldolase, collagen, enolase, and parvalbumin. The effect of temperature on the IgE-binding of the different allergens was also assessed. Furthermore, the protein extracts were tested regarding their suitability as skin prick test extracts on salmon processing workers. The results demonstrate that salmon protein extracts contain important allergens, which are sufficient to detect allergic sensitization to salmon resulting from exposure at work as well as in domestic settings.

The most common route of exposure in the occupational setting is via inhalation through upper and lower airways, which is in contrast to food exposure, where the gastrointestinal tract is the main gate of entry (41). Both exposure pathways have structural barriers between the internal and external environments as well as clearance mechanisms which affects the ability of the allergens to interact with the immune system. There is also a difference between the parts of the salmon that are involved; salmon processing workers are exposed to organs and tissues that are usually removed before eating. This includes e.g., skin, mucus, and entrails, whereas the main ingested part of salmon is muscle tissue. Tissues that are removed before eating might contain allergens and adjuvants that are relevant in the occupational setting but less so in food exposure.

The 12 kDa muscle protein parvalbumin is known as the main fish allergen. In addition to the monomeric form, parvalbumin may be present as isoforms and oligomers with varying molecular weights. When investigating our raw muscle protein extract with a commercial antibody for parvalbumin, we identified binding activity in protein bands sized ∼12, ∼28, ∼50, ∼70, ∼100, and ∼200 kDa (Figure 2D). Previous findings have identified antibody-binding activity of parvalbumin at molecular weights 12–130 kDa in cod (19, 42) and 28, 41, 47, and 49 kDa in cod, haddock, and salmon (43).

Many food allergens are heat-stable proteins, retaining their antibody binding ability even after cooking. This has also been shown for parvalbumin (44). In our study, we found that the monomeric form of parvalbumin (10–12 kDa) was stable at 100°C when heated for 5 min, but antibody-binding activity started to weaken at 30 min (Table 2). In our heated muscle extract, we found antibody reactivity at protein bands higher than the monomeric form (>12 kDa), which seems to be more susceptible for degradation at temperatures lower than 100°C. This agrees with the results of Sletten et al. (43), who demonstrated that oligomeric forms of parvalbumin seemed to be more susceptible of losing their IgE-binding capacity after processing, as opposed to the monomeric form (43). Heating, or other forms of processing such as cooking, baking, frying, canning, etc., can alter the allergenicity by changing the tertiary structure of proteins, which might make epitopes either more or less available to IgE-binding, or reduce the number of binding sites (45).

Interestingly, we did not identify parvalbumin to be present in our mucus protein extract, as verified by both immunoblotting and MS/MS analysis of the extract. Considering that parvalbumin is well known as the major fish allergen, this is an important finding. This is especially true in the occupational context, where salmon processing workers are exposed to other parts of the salmon than the muscle tissue, which is mostly the case in a domestic setting. This emphasizes the value of having well characterized skin prick test extracts, that contain allergens sufficient in detecting both food and occupational sensitization.

Collagen is another heat stable fish allergen of important clinical relevance, as demonstrated in a study by Kalic et al. where 21% of patients in the study were sensitized to this allergen (46). In the present paper, we found collagen to be mainly present in skin extracts, but it was also identified in muscle and mucus extracts. We evaluated the thermolability of collagen by heating the skin extract at various temperatures (Table 2). Immunoblotting of the heated extract showed that degradation of collagen occurs at prolonged heating (30 min) at 80°C and 100°C but is stable when heated for 5 min at the same temperatures. Collagen is generally insoluble in aqueous solutions and thus is normally not part of commercial fish allergen extracts. Our extraction protocol was based on extraction of acid-soluble collagen from salmon skin. This method has previously been shown successful in extracting the allergenic type I collagen alpha (46, 47). Additionally, we could document that the skin extract contained several isoforms of collagen, which was confirmed through immunoblotting and MS/MS (Figure 4, Supplementary Table S2).

The heat sensitive allergenic proteins aldolase and enolase were found in raw muscle and mucus extracts. Immunoblotting of raw muscle extracts revealed bands corresponding to monomeric aldolase at ∼35 and ∼40 kDa, whereas enolase showed antibody reactivity at ∼40 and ∼47 kDa. MS/MS analysis of this extract identified the presence of aldolase and enolase in all four bands that were analyzed: ∼12, ∼40, ∼50, and ∼110 kDa (Supplementary Table S3). The presence at 12 kDa might be degraded allergenic proteins, whereas the presence at 110 kDa can indicate a dimeric form. Binding at higher molecular weights can also consist of aggregates formed with other proteins, or oligomeric forms as demonstrated by Kuehn et al. (25). In our heated muscle extract, we found that antibody-binding activity corresponding to monomeric aldolase disappeared when heated at 60°C for 5 min, and for enolase at the same temperature but after 30 min (Table 2). The thermostability of aldolase and enolase has previously been assessed in a study by Ruethers et al. (48). Mass spectrometric analysis in their study showed that aldolase and enolase were absent in heated protein extracts from both salmon and catfish. It has been found that enolases from the muscle of red sea bream, pacific mackerel and carp requires divalent metal ions for their proper functioning (49). The loss of antibody-binding activity at higher temperatures might be explained by conformational changes causing insufficient binding to the cofactor. Nakagawa and Nagayama also found that aldolase in the three species displayed loss of activity following heating at 50°C (50). Another study reported on the heat labile nature of aldolase from two species of Antarctic fish (51).

The results from skin prick testing of four salmon processing workers (Table 3) demonstrate the importance of using well-characterized extracts to provide information about individual sensitization profiles. The four individuals were selected from a larger group of SPT positive salmon workers to exhibit (1) that our extracts perform as expected, with respect to not producing false-positive results and (2) that individuals might display different sensitization profiles, thus emphasizing the importance of having skin prick test extracts that contain all relevant allergens in both the domestic and occupational setting. Future research should aim to standardize allergen concentrations and eliminate the possibility of microbial presence. The next planned paper will describe more detailed findings from the use of the skin prick test extracts as well as other immunological data in the referred study population.

## Conclusion

Occupational exposure to fish differs from food exposure by the contact with mainly unprocessed or uneatable parts of the fish, as well as different routes of entry to the human body. These differences call for fitted diagnostic tools, including skin prick test extracts containing the relevant allergens in each exposure scenario. In this study we made protein extracts containing known allergens from salmon which might be of special relevance to occupationally exposed salmon workers. We show for the first time that using skin prick test extracts based on different salmon tissues is effective in detecting sensitization to thermolabile allergens, as well as allergens in uneatable parts of the salmon that may be inhaled in airborne particles or droplets or get into contact with skin. The allergy diagnostic value, however, needs to be confirmed in studies linking SPT test results to clinical outcomes in exposed populations.

## Data Availability

The original contributions presented in the study are included in the article/Supplementary Material, further inquiries can be directed to the corresponding author.

## Footnotes

^1^Upon subsequent extraction, the ultracentrifuge step was omitted due to instrument failure, and was replaced by several steps of centrifugation with the Allegra® X-15R Centrifuge.

## Supplementary material

Supplementary Figure 1 Protein content of heat-treated protein extracts from Atlantic salmon by SDS-PAGE, separated in 4-12% Bis-Tris gel. (A) Protein content in a heated muscle extract, stained with silver staining. (B) Protein content in a heated mucus extract, stained with Coomassie. (C) Protein content in a heated skin extract, stained with Coomassie. Letters in lowercase corresponds to bands that were analyzed using mass spectrometry, matching the monomeric form of the four allergens collagen: a, e, i, and j, enolase: b and f, aldolase: c and g, and parvalbumin: d and h. Extracts used were RM3, M1 and S1 from Table S1.

Supplementary Figure 2 Detection of allergens in a heated muscle extract from Atlantic salmon. Proteins were separated by SDS-PAGE in a 4-12% Bis-Tris gel and detected with commercial antibodies through immunoblotting: (A) enolase, (B) aldolase, and (C) parvalbumin. Letters in lowercase corresponds to bands that were analyzed using mass spectrometry, matching the monomeric form of the allergens: b: enolase (∼50 kDa), c: aldolase (∼40 kDa), and d: parvalbumin (∼12 kDa). Extract used was RM2 from Table S1.

Supplementary Figure 3 Detection of allergens in a heated mucus extract from Atlantic salmon. Proteins were separated by SDS-PAGE in a 4-12% Bis-Tris gel and detected with commercial antibodies through immunoblotting: (A) collagen, (B): enolase, and (C): aldolase. Letters in lowercase corresponds to bands that were analyzed using mass spectrometry, matching the monomeric form of the allergens: e: collagen (∼110 kDa), f: enolase (∼50 kDa), and G: aldolase (∼40 kDa). Extract used was M1 from Table S1.

Supplementary Figure 4 Detection of collagen in a heated skin extract from Atlantic salmon. The protein extract was separated by SDS-PAGE in a 4-12% Bis-Tris gel and detected with a commercial antibody for collagen through immunoblotting. Letters in lowercase corresponds to protein bands that were analyzed using mass spectrometry. Extract used was S1 from Table S1.
