# Supplementary figures and images for: Tissue-based skin prick test extracts from Atlantic salmon containing occupationally relevant allergens
Source: Front Allergy. 2025 Jun 23;6:1525012. doi: 10.3389/falgy.2025.1525012 (PMC12230974; doi:10.3389/falgy.2025.1525012)

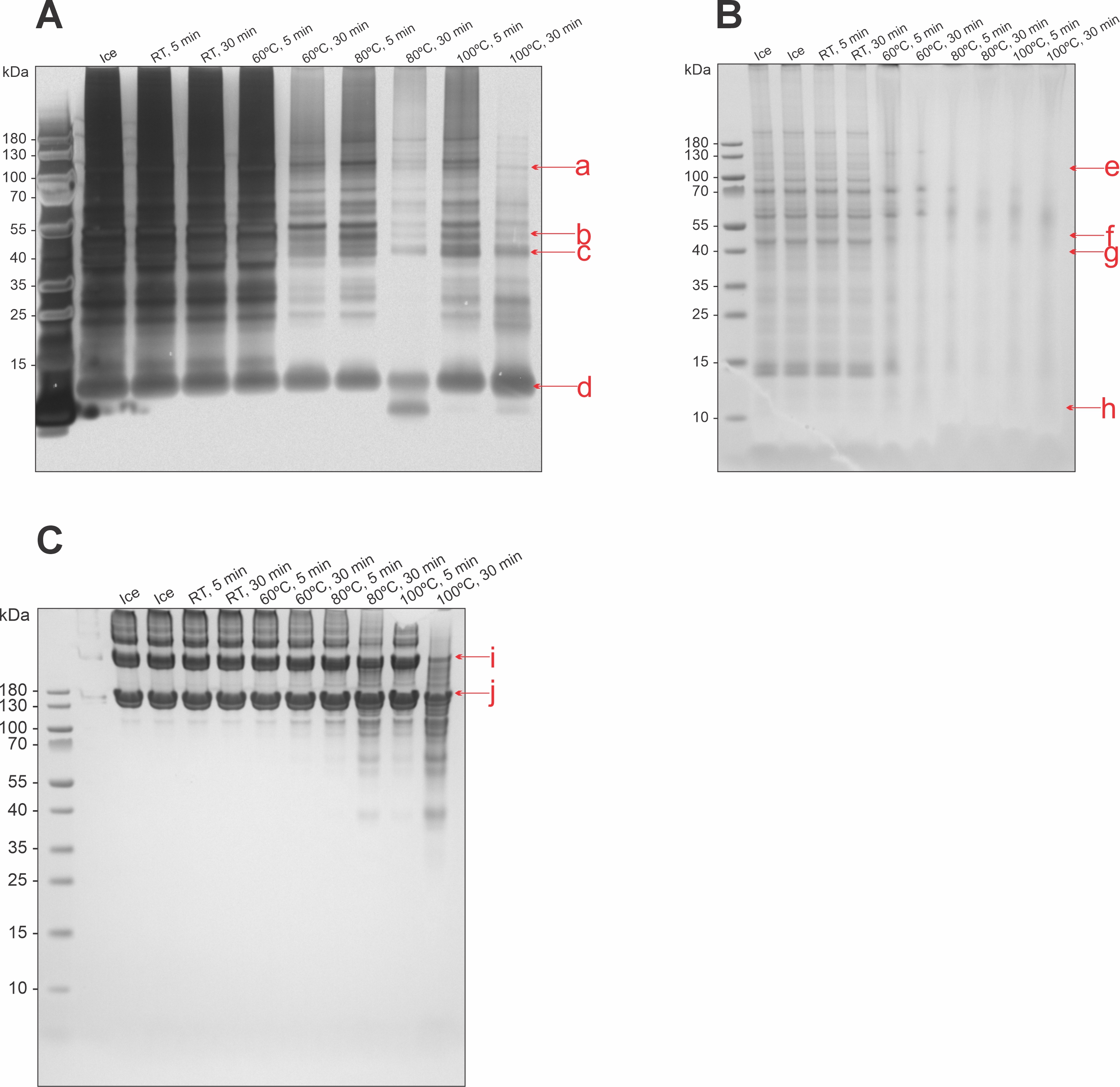

Supplement: Supplementary Figure 1 — Protein content of heat-treated protein extracts from Atlantic salmon by SDS-PAGE, separated in 4-12% Bis-Tris gel. (A) Protein content in a heated muscle extract, stained with silver staining. (B) Protein content in a heated mucus extract, stained with Coomassie. (C) Protein content in a heated skin extract, stained with Coomassie. Letters in lowercase corresponds to bands that were analyzed using mass spectrometry, matching the monomeric form of the four allergens collagen: a, e, i, and j, enolase: b and f, aldolase: c and g, and parvalbumin: d and h. Extracts used were RM3, M1 and S1 from Table S1. [file Image1.jpeg]

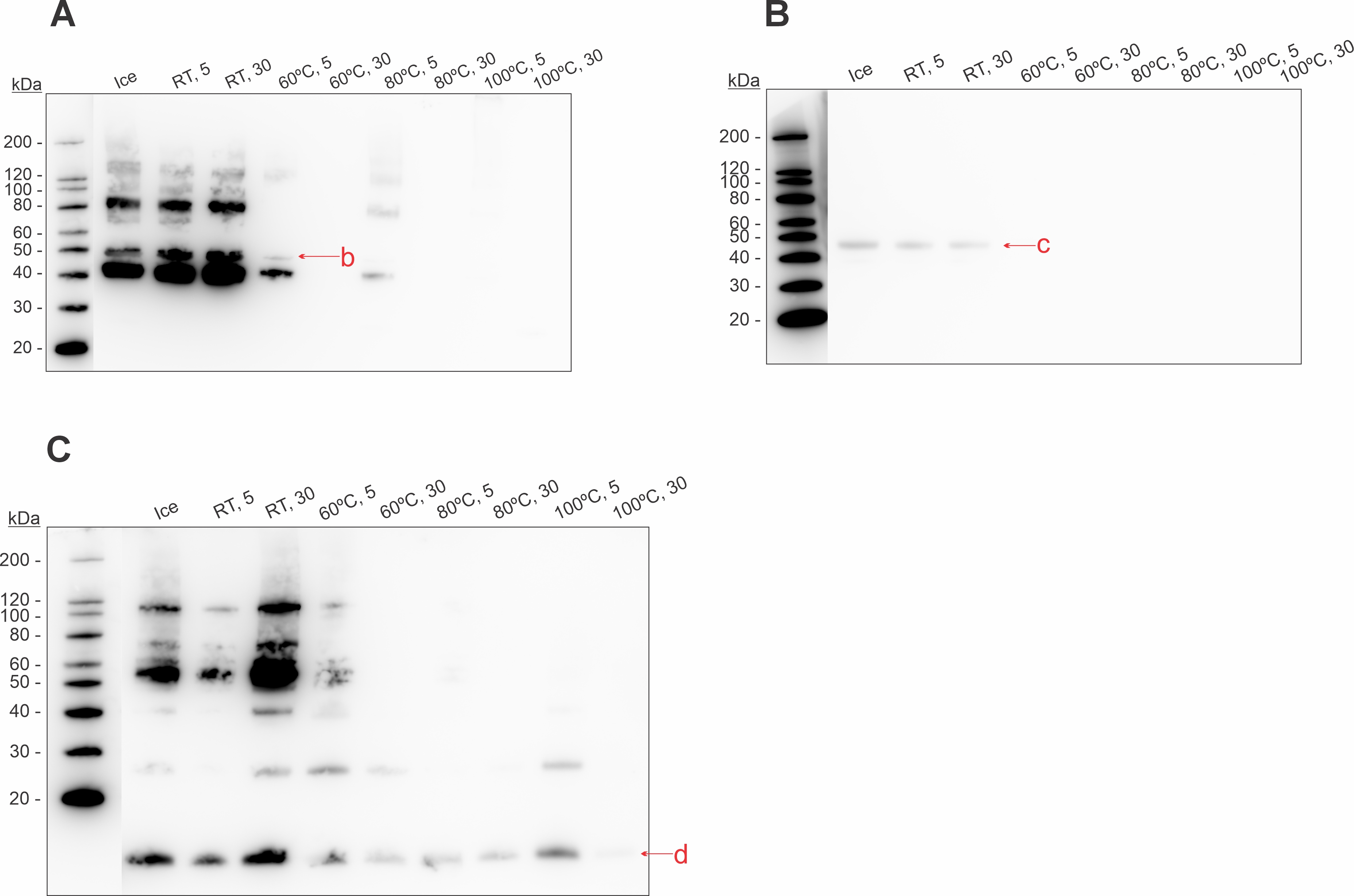

Supplement: Supplementary Figure 2 — Detection of allergens in a heated muscle extract from Atlantic salmon. Proteins were separated by SDS-PAGE in a 4-12% Bis-Tris gel and detected with commercial antibodies through immunoblotting: (A) enolase, (B) aldolase, and (C) parvalbumin. Letters in lowercase corresponds to bands that were analyzed using mass spectrometry, matching the monomeric form of the allergens: b: enolase (∼50 kDa), c: aldolase (∼40 kDa), and d: parvalbumin (∼12 kDa). Extract used was RM2 from Table S1. [file Image2.jpeg]

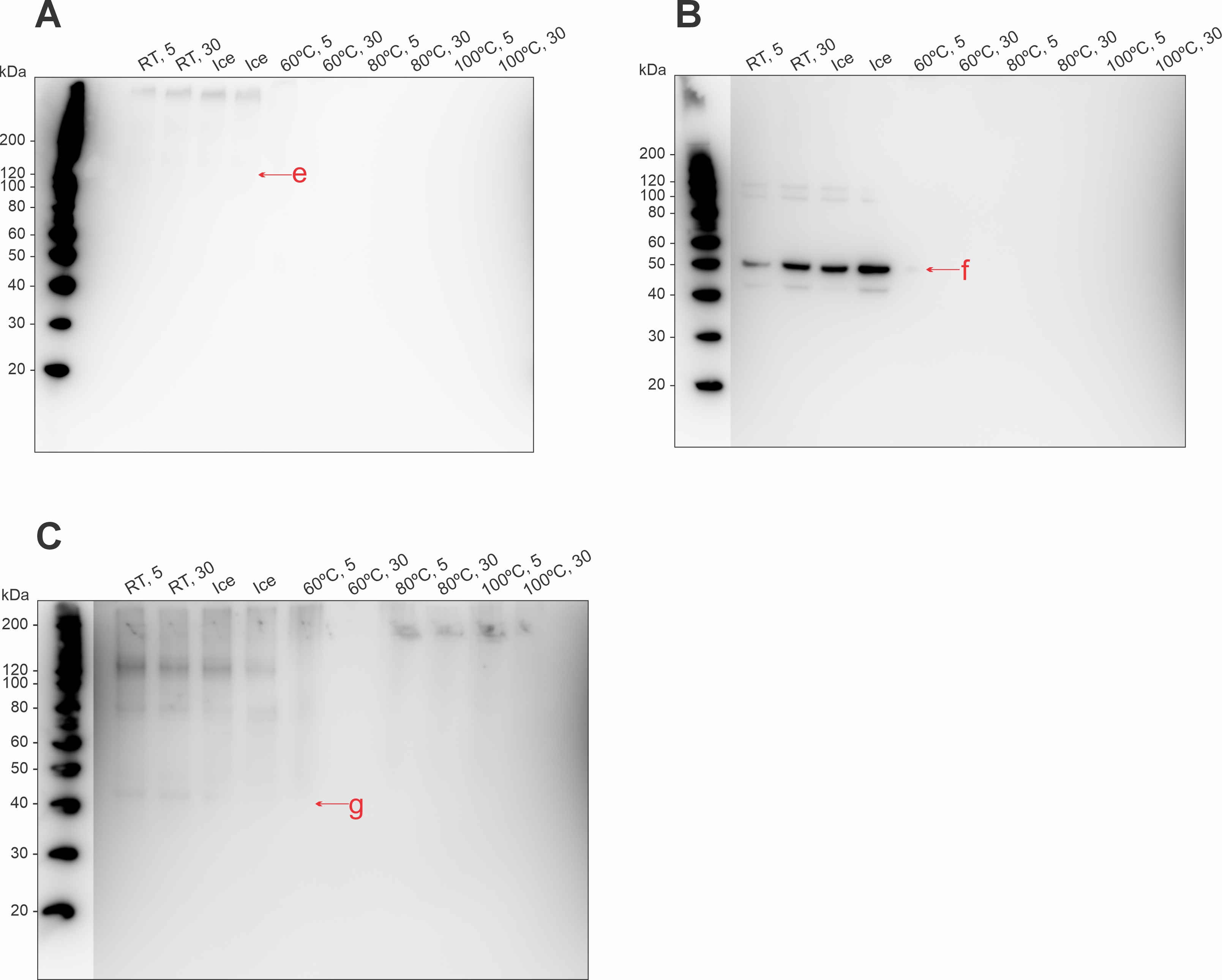

Supplement: Supplementary Figure 3 — Detection of allergens in a heated mucus extract from Atlantic salmon. Proteins were separated by SDS-PAGE in a 4-12% Bis-Tris gel and detected with commercial antibodies through immunoblotting: (A) collagen, (B): enolase, and (C): aldolase. Letters in lowercase corresponds to bands that were analyzed using mass spectrometry, matching the monomeric form of the allergens: e: collagen (∼110 kDa), f: enolase (∼50 kDa), and G: aldolase (∼40 kDa). Extract used was M1 from Table S1. [file Image3.jpeg]

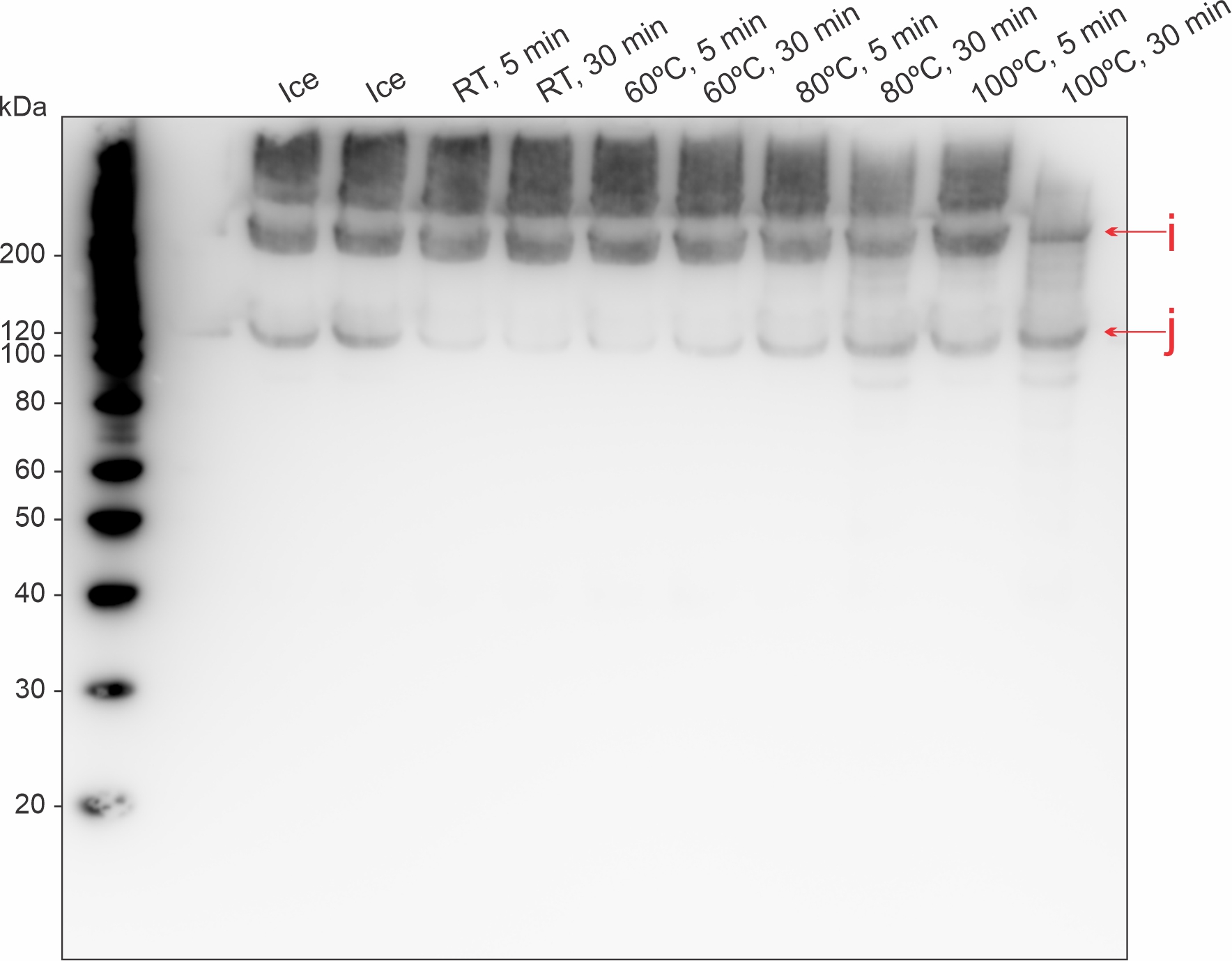

Supplement: Supplementary Figure 4 — Detection of collagen in a heated skin extract from Atlantic salmon. The protein extract was separated by SDS-PAGE in a 4-12% Bis-Tris gel and detected with a commercial antibody for collagen through immunoblotting. Letters in lowercase corresponds to protein bands that were analyzed using mass spectrometry. Extract used was S1 from Table S1. [file Image4.jpeg]
