# Supplementary material for: Tissue-based skin prick test extracts from Atlantic salmon containing occupationally relevant allergens
Source: Front Allergy. 2025 Jun 23;6:1525012. doi: 10.3389/falgy.2025.1525012 (PMC12230974; doi:10.3389/falgy.2025.1525012)
Supplement: Supplementary file 5 [file Datasheet1.docx]

**Table S1.** Protein concentration in in-house tissue extracts from raw muscle, mucus, cooked muscle, and skin, with reference to figures and tables in the manuscript. The extracts were analyzed using the QuantiPro™ BCA Assay Kit (Sigma-Aldrich®).

| **Extract** | **Protein concentration (μg/ml)** | **Reference in paper** |
| --- | --- | --- |
| Raw muscle 1 (RM1)* | 12920 | Figure 1, Table 3 |
| Raw muscle 2 (RM2) ^ᵻ^ | 52 | Figure 2, Supplementary Figure 2, Table 2 |
| Raw muscle 3 (RM3)^ᵻ^ | 20,63 | Supplementary Figure 1A |
| Mucus 1 (M1)* ^ᵻ^ | 6523 | Figure 1, Table 2, Table 3 Supplementary Figure 1B, Supplementary Figure 3 |
| Mucus 2 (M2) | 196,57 | Figure 3 |
| Cooked muscle 1 (CM1)* | 9373 | Figure 1, Table 3 |
| Skin 1 (S1)* ^ᵻ^ | 6577 | Figure 1, Table 2, Table 3 Supplementary Figure 1C, Supplementary Figure 4 |
| Skin 2 (S2) | 58 | Figure 4 |

*: extracts that were used as SPT extracts in SHInE field work.

^ᵻ^ : extracts that were used as basis for heated extracts, BCA not done after heat treatment.

**Table S2.** MS/MS identification of WHO/IUIS registered allergen in skin tissue extract from Atlantic salmon.

| **Mw of protein band** | **Identified protein** | **Accession** | **Theoretical Mw (kDa)** | **Coverage (%)** | **Peptides identified by MS/MS** | **# PSMs** |
| --- | --- | --- | --- | --- | --- | --- |
| **230 kDa** | Collagen alpha-1(I) chain-like | A0A1S3R8F9 | 136,8 | 10 | 8 | 11 |
|  | Collagen alpha-1(I) chain | A0A1S3S6G4 | 136,9 | 7 | 6 | 9 |
|  | Collagen alpha-2(I) chain isoform X1 | A0A1S3Q205 | 126,9 | 9 | 9 | 9 |
|  | Collagen alpha-1(I) chain-like | A0A1S3SM10 | 138,1 | 6 | 6 | 5 |
|  | Collagen alpha-1(I) chain-like | A0A1S3Q7E3 | 137,4 | 5 | 5 | 5 |
|  | Collagen alpha-3(VI) chain-like | A0A1S3QZQ5 | 44,1 | 6 | 6 | 2 |
|  | Collagen alpha-1(XVII) chain-like isoform X1 | A0A1S3L656 | 158,6 | 1 | 1 | 1 |
| **200 kDa** | Collagen alpha-1(I) chain-like | A0A1S3R8F9 | 136,8 | 16 | 12 | 18 |
|  | Collagen alpha-1(I) chain | A0A1S3S6G4 | 136,9 | 18 | 13 | 17 |
|  | Collagen alpha-2(I) chain isoform X1 | A0A1S3M538 | 126,9 | 12 | 11 | 11 |
|  | Collagen alpha-2(I) chain isoform X1 | A0A1S3Q205 | 126,9 | 12 | 11 | 11 |
|  | Collagen alpha-1(I) chain-like | A0A1S3Q7E3 | 137,4 | 5 | 5 | 5 |
|  | Collagen alpha-1(I) chain-like | A0A1S3SM10 | 138,1 | 5 | 4 | 4 |
|  | Collagen alpha-3(VI) chain-like | A0A1S3QZQ5 | 44,1 | 5 | 2 | 2 |
|  | Collagen alpha-1(XVII) chain-like isoform X1 | A0A1S3L656 | 158,6 | 1 | 1 | 1 |
| **180 kDa** | Collagen alpha-2(I) chain isoform X1 | A0A1S3M538 | 126,9 | 8 | 9 | 9 |
|  | Collagen alpha-2(I) chain isoform X1 | A0A1S3Q205 | 126,9 | 7 | 8 | 8 |
|  | Collagen alpha-1(I) chain-like | A0A1S3Q7E3 | 137,4 | 6 | 6 | 6 |
|  | Collagen alpha-1(I) chain-like | A0A1S3R8F9 | 136,8 | 3 | 3 | 3 |

**Table S3.** MS/MS identification of WHO/IUIS registered allergens in raw muscle extract from Atlantic salmon.

| **Mw of protein band** | **Identified protein** | **Accession** | **Theoretical Mw (kDa)** | **Coverage (%)** | **Peptides identified by MS/MS** | **# PSMs** |
| --- | --- | --- | --- | --- | --- | --- |
| **110 kDa** | 2-phospho-D-glycerate hydro-lyase | A0A1S2WZE3 | 47,3 | 34 | 13 | 53 |
|  | 2-phospho-D-glycerate hydro-lyase | A0A1S2X522 | 47,2 | 31 | 12 | 53 |
|  | Collagen alpha-2(I) chain isoform X1 | A0A1S3M538 | 126,9 | 1 | 2 | 2 |
|  | Creatine kinase | B5DGP0 | 42,9 | 45 | 18 | 172 |
|  | Creatine kinase | B5DGG5 | 42,7 | 42 | 16 | 208 |
|  | Creatine kinase | B5DGP2 | 42,9 | 41 | 17 | 141 |
|  | Creatine kinase | A0A1S3KND3 | 47,2 | 12 | 5 | 10 |
|  | Fructose-bisphosphate aldolase A | B5DGM7 | 39,5 | 57 | 20 | 209 |
|  | Fructose-bisphosphate aldolase | A0A1S2X3Z6 | 39,7 | 55 | 18 | 174 |
|  | Triosephosphate isomerase | A0A1S3P5Q0 | 26,5 | 51 | 11 | 47 |
|  | Triosephosphate isomerase | B5XB51 | 26,6 | 15 | 3 | 9 |
| **50 kDa** | 2-phospho-D-glycerate hydro-lyase | B5X1B5 | 47 | 39 | 14 | 122 |
|  | 2-phospho-D-glycerate hydro-lyase | A0A1S2WZE3 | 47,3 | 77 | 32 | 710 |
|  | 2-phospho-D-glycerate hydro-lyase | A0A1S2X522 | 47,2 | 73 | 31 | 723 |
|  | Creatine kinase | B5DGP0 | 42,9 | 42 | 15 | 73 |
|  | Creatine kinase | B5DGG5 | 42,7 | 36 | 13 | 77 |
|  | Creatine kinase | B5DGP2 | 42,9 | 39 | 14 | 62 |
|  | Creatine kinase | A0A1S3KND3 | 47,2 | 10 | 4 | 9 |
|  | Fructose-bisphosphate aldolase A | B5DGM7 | 39,5 | 54 | 21 | 154 |
|  | Fructose-bisphosphate aldolase | A0A1S2X3Z6 | 39,7 | 53 | 20 | 127 |
|  | Parvalbumin beta 1-like | A0A1S3S2P0 | 12,4 | 7 | 1 | 2 |
|  | Triosephosphate isomerase | A0A1S3P5Q0 | 26,5 | 19 | 4 | 6 |
| **40 kDa** | 2-phospho-D-glycerate hydro-lyase | A0A1S2WZE3 | 47,3 | 17 | 6 | 15 |
|  | 2-phospho-D-glycerate hydro-lyase | A0A1S2X522 | 47,2 | 17 | 6 | 17 |
|  | Creatine kinase | B5DGP0 | 42,9 | 53 | 20 | 962 |
|  | Creatine kinase | B5DGG5 | 42,7 | 54 | 21 | 1092 |
|  | Creatine kinase | B5DGP2 | 42,9 | 53 | 20 | 938 |
|  | Creatine kinase | A0A1S3KND3 | 47,2 | 19 | 7 | 27 |
|  | Creatine kinase | A0A1S3SNZ4 | 34,9 | 10 | 2 | 26 |
|  | Fructose-bisphosphate aldolase A | B5DGM7 | 39,5 | 76 | 33 | 1538 |
|  | Fructose-bisphosphate aldolase | C0H9I1 | 39,7 | 51 | 15 | 379 |
|  | Fructose-bisphosphate aldolase | A0A1S2X3Z6 | 39,7 | 76 | 31 | 1313 |
|  | Triosephosphate isomerase | A0A1S3P5Q0 | 26,5 | 46 | 8 | 17 |
| **12 kDa** | 2-phospho-D-glycerate hydro-lyase | A0A1S2WZE3 | 47,3 | 9 | 4 | 14 |
|  | 2-phospho-D-glycerate hydro-lyase | A0A1S2X522 | 47,2 | 9 | 4 | 8 |
|  | Creatine kinase | B5DGP0 | 42,9 | 2 | 1 | 1 |
|  | Fructose-bisphosphate aldolase A | B5DGM7 | 39,5 | 28 | 12 | 40 |
|  | Fructose-bisphosphate aldolase | A0A1S2X3Z6 | 39,7 | 24 | 9 | 37 |
|  | Parvalbumin beta 1-like | A0A1S3S2P0 | 12,4 | 74 | 7 | 193 |
|  | Parvalbumin beta 2 | Q91483 | 11,4 | 35 | 4 | 15 |
|  | Triosephosphate isomerase | A0A1S3P5Q0 | 26,5 | 19 | 4 | 4 |

**Table S4.** Identification of WHO/IUIS registered allergens in mucus extract from Atlantic salmon.

| **Mw of protein band** | **Identified protein** | **Accession** | **Theoretical Mw (kDa)** | **Coverage (%)** | **Peptides identified by MS/MS** | **# PSMs** |
| --- | --- | --- | --- | --- | --- | --- |
| **110 kDa** | Collagen alpha-3(VI) chain-like | A0A1S3R2W7 | 30,8 | 3 | 1 | 1 |
| **50 kDa** | 2-phospho-D-glycerate hydro-lyase | B5X1B5 | 47 | 8 | 4 | 4 |
|  | 2-phospho-D-glycerate hydro-lyase | A0A1S2WZE3 | 47,3 | 6 | 2 | 2 |
|  | 2-phospho-D-glycerate hydro-lyase | A0A1S3LBJ0 | 47,1 | 6 | 3 | 3 |
|  | Collagen alpha-3(VI) chain-like | A0A1S3PM55 | 264,1 | 9 | 8 | 8 |
|  | Creatine kinase | B5DGP0 | 42,9 | 25 | 8 | 10 |
|  | Creatine kinase | B5DGG5 | 42,7 | 21 | 6 | 7 |
|  | Creatine kinase | B5X0S0 | 42,7 | 3 | 1 | 1 |
|  | Creatine kinase | A0A1S3SNZ4 | 34,9 | 12 | 3 | 4 |
|  | Fructose-bisphosphate aldolase A | B5DGM7 | 39,5 | 35 | 7 | 11 |
|  | Fructose-bisphosphate aldolase | C0H9I1 | 39,7 | 39 | 10 | 15 |
|  | Fructose-bisphosphate aldolase | O73866 | 39,2 | 23 | 6 | 7 |
|  | Fructose-bisphosphate aldolase | A0A1S2X3Z6 | 39,7 | 27 | 6 | 10 |
|  | Fructose-bisphosphate aldolase | A0A1S3RSZ1 | 38,9 | 15 | 4 | 4 |
| **40 kDa** | 2-phospho-D-glycerate hydro-lyase | B5X1B5 | 47 | 12 | 4 | 4 |
|  | 2-phospho-D-glycerate hydro-lyase | A0A1S2WZE3 | 47,3 | 9 | 3 | 3 |
|  | Collagen alpha-3(VI) chain-like | A0A1S3PM55 | 264,1 | 8 | 9 | 9 |
|  | Creatine kinase | B5DGP0 | 42,9 | 14 | 4 | 4 |
|  | Creatine kinase | B5DGP0 | 42,7 | 14 | 5 | 6 |
|  | Creatine kinase | A0A1S3SNZ4 | 34,9 | 2 | 1 | 1 |
|  | Creatine kinase | A0A1S3PDD7 | 39,5 | 5 | 1 | 1 |
|  | Fructose-bisphosphate aldolase | C0H9I1 | 39,7 | 29 | 7 | 9 |
|  | Fructose-bisphosphate aldolase | A0A1S2X3Z6 | 39,7 | 23 | 5 | 6 |
|  | Triosephosphate isomerase | B5XB51 | 26,6 | 6 | 1 | 1 |
|  | Tropomyosin alpha-1 chain-like isoform X1 | A0A1S3MM38 | 32,7 | 4 | 1 | 1 |
| **12 kDa** | 2-phospho-D-glycerate hydro-lyase | B5X1B5 | 47 | 2 | 1 | 1 |
|  | Collagen alpha-3(VI) chain-like | A0A1S3QZQ5 | 44,1 | 6 | 2 | 2 |
